# Immunoproteomic analysis of the protein repertoire of unsporulated *Eimeria tenella* oocysts

**DOI:** 10.1051/parasite/2017047

**Published:** 2017-12-01

**Authors:** Zhenchao Zhang, Shuai Wang, Charles Li, Liheng Liu

**Affiliations:** 1 School of Basic Medical Sciences, Xinxiang Medical University, Xinxiang, Henan, 453003 PR China; 2 Animal Biosciences and Biotechnology Laboratory, Agricultural Research Service, United States Department of Agriculture, Beltsville, MD 20705 USA; 3 Jiangxi Provincial Key Laboratory for Animal Health, College of Animal Science and Technology, Jiangxi Agricultural University, No.1101 Zhimin Avenue, Economic and Technological Development District, Nanchang 330045, Jiangxi PR China

**Keywords:** *E. tenella*, unsporulated oocysts, immunoproteome

## Abstract

The apicomplexan protozoans *Eimeria* spp. cause coccidioses, the most common intestinal diseases in chickens. Coccidiosis is associated with significant animal welfare issues and has a high economic impact on the poultry industry. Lack of a full understanding of immunogenic molecules and their precise functions involved in the *Eimeria* life cycles may limit development of effective vaccines and drug therapies. In this study, immunoproteomic approaches were used to define the antigenic protein repertoire from the total proteins of unsporulated *Eimeria tenella* oocysts. Approximately 101 protein spots were recognized in sera from chickens infected experimentally with *E. tenella*. Forty-six spots of unsporulated oocysts were excised from preparative gels and identified by matrix-assisted laser desorption ionization time-of-flight MS (MALDI-TOF-MS) and MALDI-TOF/TOF-MS. For unsporulated oocysts, 13 known proteins of *E. tenella* and 17 homologous proteins to other apicomplexan or protozoan parasites were identified using the ‘Mascot’ server. The remaining proteins were searched against the *E. tenella* protein sequence database using the ‘Mascot in-house’ search engine (version 2.1) in automated mode, and 12 unknown proteins were identified. The amino acid sequences of the unknown proteins were searched using BLAST against non-redundant sequence databases (NCBI), and 9 homologous proteins in unsporulated oocyst were found homologous to proteins of other apicomplexan parasites. These findings may provide useful evidence for understanding parasite biology, pathogenesis, immunogenicity and immune evasion mechanisms of *E. tenella*.

## Abstract (fr)

Les protozoaires Apicomplexa *Eimeria* spp. causent les coccidioses, les maladies intestinales les plus communes chez les poulets. La coccidiose est un problème majeur de bien-être animal et a un fort impact économique sur l'industrie de la volaille. Le manque de compréhension complète des molécules immunogènes et de leurs fonctions précises impliquées dans les cycles de vie d'*Eimeria* peut limiter le développement de vaccins efficaces et de pharmacothérapies. Dans cette étude, des approches immunoprotéomiques ont été utilisées pour définir le répertoire des protéines antigéniques à partir des protéines totales des oocystes non sporulés d'*Eimeria tenella*. Environ 101 taches protéiques ont été reconnues dans des sérums de poulets infectés expérimentalement par *E. tenella*. Quarante-six taches d'oocystes non sporulés ont été excisées à partir de gels et identifiées par MALDI-TOF-MS et MALDI-TOF/TOF-MS par désorption laser assistée par matrice. Pour les oocystes non sporulés, 13 protéines connues d'E*. tenella* et 17 protéines homologues à d'autres parasites Apicomplexa ou protozoaires ont été identifiées en utilisant le serveur “Mascot”. Les protéines restantes ont été recherchées dans la base de données de séquences protéiques d'*E. tenella* en utilisant le moteur de recherche “Mascot in-house” (version 2.1) en mode automatisé, et 12 protéines inconnues ont été identifiées. Les séquences d'acides aminés des protéines inconnues ont été recherchées en utilisant BLAST contre des bases de données de séquences non redondantes (NCBI), et 9 protéines homologues des oocystes non-sporulés ont été trouvées homologues à des protéines d'autres parasites Apicomplexa. Ces résultats peuvent fournir des éléments utiles pour comprendre la biologie parasitaire, la pathogenèse, l'immunogénicité et les mécanismes d'évasion immunitaire d'*E. tenella*.

## Introduction

*E. tenella* is an apicomplexan protozoan parasite and one of the etiological agents of coccidiosis in poultry. Coccidiosis is the most common intestinal disease in poultry and has considerable welfare and economic implications in the poultry industry [7]. At present, coccidiosis is mainly controlled by prophylactic medication via the use of in-feed anticoccidial drugs known as coccidiostats [29]. However, the excessive use of these drugs has led to drug-resistance and involves a food safety concern. Novel approaches are urgently needed for the effective control of coccidiosis [9,31].

Understanding the pathogenesis is important for designing effective strategies to control coccidiosis. When released into the environment, the unsporulated oocysts of *E. tenella* undergo meiosis upon contact with oxygen and moisture [2]. Metabolism is not constant throughout the process of sporulation. Wilson and Fairbairn (1961) found that polysaccharides were utilized initially, but later lipid metabolism played a greater role [38]. Oxygen consumption and the relationship with different stages in sporulation were also studied by Wagenbach and Burns (1969) [35]. Extensive studies were performed on the sporulation processes of *E. tenella* oocysts by Yvoré and Coudert (1972) [10]. They separated the sporulation process into four different morphological stages related to cumulative oxygen consumption and found that the quantity of oxygen necessary at a given stage or completion of sporulation was constant and independent of temperature. Wilson & Fairbainz [38] and Wang [36] showed that respiration during sporulation initially occurred at a higher rate, but later at a lower rate when the sporulation was completed. Moreover, enzyme turnover was also studied in the unsporulated oocyst stage. Some enzymes were identified in unsporulated oocysts, but their levels decreased during the late phase of sporulation [37]. Therefore, the processing of sporulated oocysts is very important for the spread of coccidiosis in chickens. Whether the immune system of the host recognizes these key molecules in the sporulation process may affect the formation of sporulated oocysts, and could provide clues for controlling the spread of coccidiosis in chickens.

Proteomic expression studies have been proposed as a powerful method to discover new drug targets for parasitic diseases [1,33]. With advances in *Eimeria* genomics, the protein repertoire of the microneme organelles and refractile body of E. tenella has been identified via proteomic approaches [7,11]. Some protein repertoires of microneme secretory organelles were identified in micronemes. Immunoproteomics was also used to identify immunogenic molecules and pathogenicity factors in the sporozoite stage of *E. tenella*. Approximately 50 spots of the 130 analyzed spots were defined as antigens on the basis of the anti-E. tenella chicken sera used [11]. Liu et al. studied the whole proteins of the second-generation merozoite of *E. tenella* by two-dimensional gel electrophoresis (2-DE) and western blotting using sera isolated from chickens infected experimentally with *E. tenella*, and approximately 640 spots were detected on the proteome map, while 85 spots were recognized [24].

Mass spectrometry (MS)-based proteomic profiling and protein identification is a powerful tool for the discovery of new disease biomarkers. Among the MS platforms, matrix-assisted laser desorption/ionization time-of-flight/time-of-flight (MALDI-TOF/TOF) MS offers high sample throughput and flexibility in qualitative and quantitative analysis of amino and organic acids. In order to detect the immunogenic proteins of unsporulated oocysts, an immunoproteomic approach coupled with MALDI-TOF/TOF-MS was used in this study to identify the antigenic proteins from the total proteins of unsporulated oocysts. Defining the immunogenic proteins will make it possible to design potential vaccine targets to effectively control coccidiosis, and these results could also enrich the knowledge on the immunogenic proteins of unsporulated oocysts.

## Materials and methods

### Ethics statement

The Ethics Committee of Jiangxi Agricultural University approved the animal protocol for this study (protocol number P-2013-03). All the procedures involving animals in this study were carried out in accordance with The Care and Use Guidelines of Experimental Animals established by the Ministry of Agriculture of China.

### Parasite collection

*E. tenella* (Nanchang strain) were maintained by passage through coccidia-free two-week-old chickens (Sanhuang Breeding Farm, Nanchang, Jiangxi, China). Unsporulated oocysts were obtained by passing the cecal contents through a 100-mesh sieve at 7 days post-infection, and the oocysts were then sterilized using 20% sodium hypochlorite and washed several times in tri-distilled water. All procedures were performed on ice. After centrifugation at 5,000 rpm for 5 min at 4°C, oocysts were stored in liquid nitrogen, as described elsewhere [32].

### Protein sample preparation

Unsporulated oocysts were broken down in lysis buffer (7 M urea, 2 M thiourea, 4% CHAPS, 40 mM dithiothreitol (DTT), 0.2% Bio-Lyte 3-10 ampholytes and 1 mM PMSF) by the ultrasonic approach in an ice bath (200 W, work time 5s, interval time 10s, 100 cycles). The content was centrifuged at 15,000 rpm for 2 min at 4°C, and the soluble proteins were cleaned with the 2D-cleanup kit and quantified using Plus One TM2-D Quant Kit (Amersham Pharmacia Biotech, Little Chalfont, UK).

### Two-dimensional electrophoresis (2-DE)

*Isoelectric focusing (IEF).* The pellet of proteins was dissolved in IPG rehydration/sample buffer (8 M urea, 2% CHAPS, 50 mM dithiothreitol (DTT), 0.2% Bio-Lyte 3-10 ampholytes, 0.001% bromophenol blue; BioRad, Hercules, CA, USA) by incubating for at least 1h at room temperature and centrifuged at 15,000 rpm for 15 min at room temperature to remove undissolved materials. 320 µL of 600 µg protein were adsorbed onto a 17 cm Immobiline DryStrip (IPG, pH range 3-10; BioRad) and IEF was performed in the BioRad PROTEAN IEF cell. Twelve hours of positive rehydration preceded zone electrophoresis at 20°C under the following conditions: S1 250 V, 1h; S2 1000 V 1h; S3 4000 V 2.5 h; and S4 8000 V 50000 V.h. Strips could be used immediately for the second dimension electrophoresis, or stored at −80 °C for a short period.

*Sodium dodecyl sulfate polyacrylamide gel electrophoresis (SDS-PAGE).* Prior to SDS-PAGE, each IPG strip was incubated in 6 mL of equilibration buffer I (375 mM Tris-HCl (pH 8.8), 6 M urea, 2% SDS, 2% DTT; BioRad) for 15 min and in 6 mL of equilibration buffer II (375 mM Tris-HCl (pH 8.8), 6 M urea, 2.5% iodoracetamide, 2% SDS; BioRad) for an additional 15 min. IPG strips and SDS-PAGE molecular weight standards (11–170 kDa; Fermentas Inc., Waltham, MA, USA) were loaded into homogeneous 12.5% polyacrylamide gels and sealed with 1% agarose solution. A Tris-glycine buffer system was used, as described by Laemmli (1970). Electrophoresis was performed in two steps at 16 °C: 3 w/gel for 45 min and 15 w/gel until the tracking dye reached the bottom of the gels. One gel was stained with Coomassie Brilliant Blue (CBB) G-250 and another was electrophoretically transferred onto a polyvinylidene fluoride (PVDF) membrane (Amersham Pharmacia Biotech) for western-blotting.

*Analysis of the stained gel.* The stained gels were scanned, and images were then analyzed using ImageMaster™ 2D Platinum Software (Version 5.0, Swiss Institute of Bioinformatics, Geneva, Switzerland) for spot detection, quantification, as well as comparison and analyses.

### Immunoblot analysis

*Preparation of immunized sera.* Chickens reared in coccidian-free conditions until 2 weeks of age were orally infected with 1 × 10^4^ sporulated oocysts of *E. tenella* per chicken. At 3 days post-first infection, the chickens were challenged four times at 3-day intervals with 5,000 sporulated oocysts per chicken. Ten negative control birds were maintained under the same conditions in an adjacent room and were inoculated by gavage with distilled water only. At 5 weeks after the last infection, blood samples were obtained from chickens by cardiac puncture. The blood was allowed to clot for 1h at 37 °C, and then overnight at 4 °C. After centrifugation at 4,000 rpm for 10 min, serum was aliquoted in 1 mL vials and stored at −20 °C until use [24].

*Western blotting.* After SDS-PAGE, proteins were transferred from the 2-DE gel onto a polyvinylidene difluoride (PVDF) membrane with blotting buffer (39 mM glycine, 48 mM Tris-base, 20% methanol, and 0.037% SDS) using a semidry blotting apparatus (TE77, Amersham Pharmacia Biotech). Electrotransfer was carried out for 2h with a current/area of 0.70 mA/cm^2^. The membrane was blocked with 5% w/v skim milk in phosphate-buffered saline (PBS) (pH 7.4) containing 0.05% Tween-20 (PBST) for 2h at room temperature, incubated with chicken serum (1:100 dilution) for 1h at room temperature and subsequently washed with PBST. It was then incubated with a horseradish peroxidase-conjugated anti-chicken IgG (a dilution of 1:2000, Bio-Rad) for 1h at room temperature. After washing with PBST, the membrane was incubated with 50 mM Tris-HCl buffer (pH 7.4) and developed with 3,3'-diaminobenzidine (DAB, Sigma, St Louis, MO, USA) substrate until optimum color development was observed. The anti-*E. tenella* serum was used to detect the immunogenic spots and the normal chicken serum was used to test another blotting membrane as a negative control.

*Spot excision and in-gel trypsin digestion.* After Western blotting, the matched protein spots on the gel were identified by comparison with the molecular size ladder using a gel documentation system (Gene Genius, Syngene, Frederick, MD, USA) and related software (GeneSnap 6.08 and GeneTools, Syngene). The matched protein spots were manually excised from the gel, destained with 30% acetonitrile (ACN) /100 mM NH_4_HCO_3_ until the gels were completely destained. The gels were dried in a vacuum centrifuge. The in-gel proteins were reduced with dithiothreitol (10 mM DTT/ 100 mM NH_4_HCO_3_) for 30 min at 56 °C, then alkylated with iodoacetamide (200 mM IAA/100 mM NH_4_HCO_3_) in the dark at room temperature for 30 minutes. Gel pieces were briefly rinsed with 100 mM NH_4_HCO_3_ and ACN, respectively. Gel pieces were digested overnight in 12.5 ng/µL trypsin in 25 mM NH_4_HCO_3_. The peptides were extracted three times with 60% ACN/0.1% trifluoroacetic acid (TFA). The extracts were pooled and dried completely using a vacuum centrifuge.

### MS analysis of protein spots and database searches

MS data for protein identification were obtained by using a MALDI-TOF/TOF instrument (5800 proteomics analyzer; Applied Biosystems, Foster City, CA, USA). Instrument parameters were set using the 4000 Series Explorer software (Applied Biosystems). The MS spectra were recorded in reflector mode in a mass range from 800 to 4000 with a focus mass of 2000. CalMix5 standard for MS was used to calibrate the instrument (ABI 4700 Calibration Mixture). For one main MS spectrum, 25 subspectra with 125 shots per subspectrum were accumulated using a random search pattern. For MS calibration, autolysis peaks of trypsin ([M + H] + 842.5100 and 2,211.1046) were used as internal calibrates, and up to 10 of the most intense ion signals were selected as precursors for MS/MS acquisition, excluding the trypsin autolysis peaks and the matrix ion signals. In MS/MS positive ion mode, 50 subspectra with 50 shots per subspectrum for one main MS spectrum were accumulated using a random search pattern. Parameters were set up as follows: Collision energy as 2 kV, collision gas as air, and default calibration as Glu1-Fibrino-peptide B ([M + H] + 1,570.6696) spotted onto Cal 7 positions of the MALDI target. Combined peptide mass fingerprinting PMF and MS/MS queries were performed by using the MASCOT search engine 2.2 (Matrix Science, Ltd.) embedded into GPS-Explorer Software 3.6 (Applied Biosystems) on the *Eimeria* database of Uniprot and NCBI, with the following parameter settings: 100 ppm mass accuracy, trypsin cleavage one missed cleavage allowed, carbamido methylation set as fixed modification, oxidation of methionine allowed as variable modification, MS/MS fragment tolerance was set to 0.4 Da. A GPS Explorer protein confidence index ≥ 95% was used for further manual validation.

## Results

### Two-dimensional gel electrophoresis of unsporulated oocyst proteins

**Figure 1:**
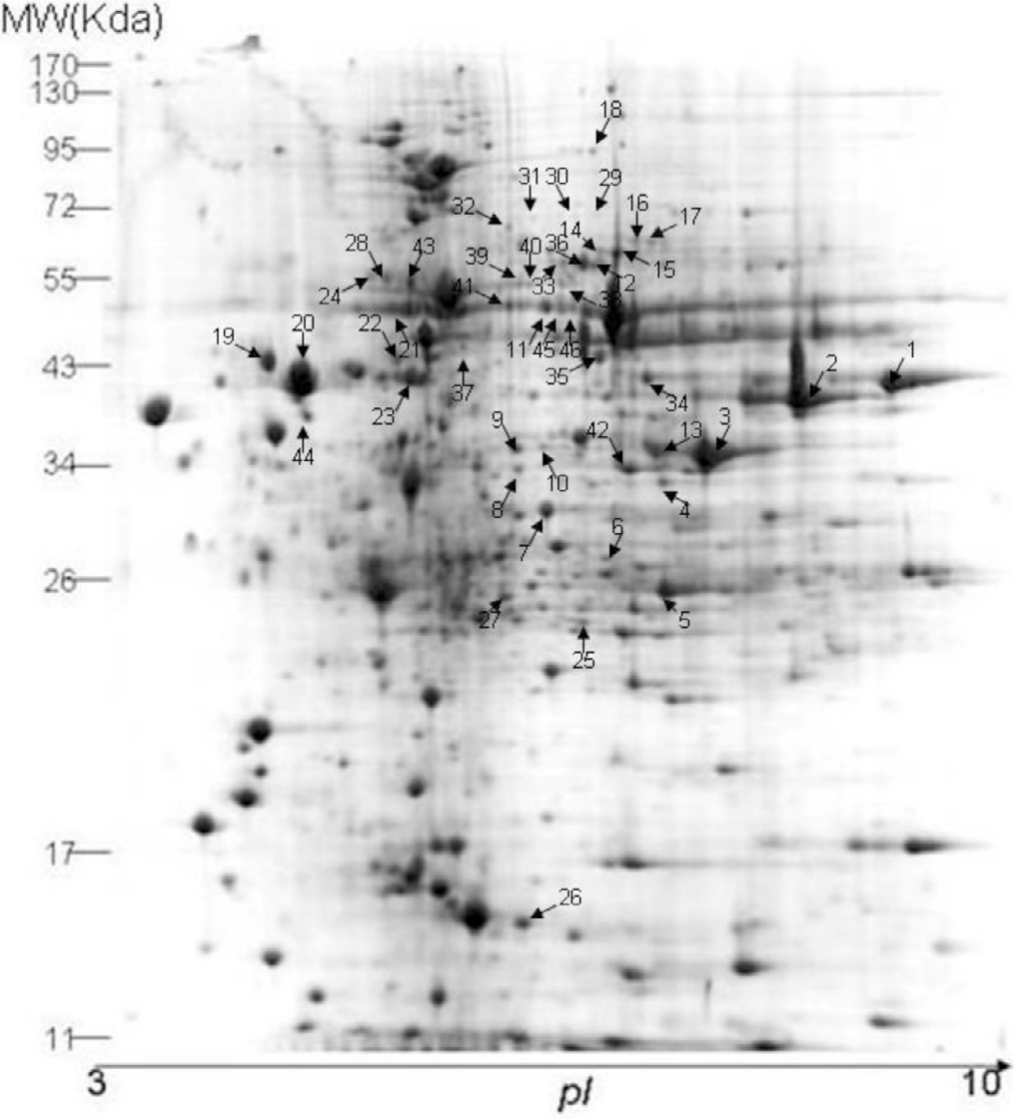
Identification of proteins of unsporulated *E. tenella* oocysts by 2-D gel electrophoresis. Isoelectro-focusing was performed with 600 μg protein for 50 kVh using a pI 3-10 strip. SDS-PAGE was performed on a 12.5% gel and then stained with CBB. Numbers are used to mark the spots analyzed.

About 600 µg/gel soluble proteins of unsporulated oocysts were separated by 2-DE over an isoelectric point (pI) range of 3-10 (IPG linear gradient). CBB G-250 stained gels revealed about 656 spots of unsporulated oocysts according to the protocol described by Candiano et al. [8]. The molecular weights of most proteins were between 11 and 130 kDa (Fig. 1).

### Analysis and identification of immunogenic proteins of unsporulated oocysts via the NCBI database

**Figure 2:**
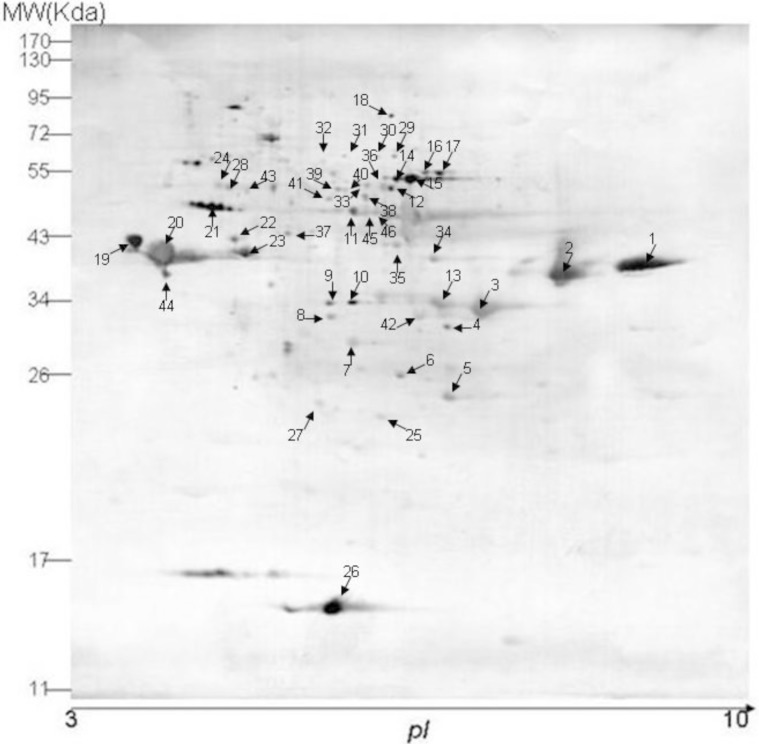
Immunoblot profiling of unsporulated *E. tenella* oocysts using chicken anti-*E. tenella* serum.

The western blot profiling of 2-DE gels is shown in Figure 2. Approximately 101 spots were observed on the PVDF membrane (Fig. 2). Among them, 46 matched closely to the protein spots and were characterized by MS. No spots were detected with normal chicken serum. Data were compared to those in the NCBI database using the ‘Mascot’ server. The probability score for the match, molecular weight (MW), pI, number of peptide matches and the sequence coverage were used for confidence of spot identification. The results are summarized in Table 1.

**Table 1:** Identification of unsporulated *Eimeria tenella* oocyst proteins in non-redundant sequence databases (NCBI) using data from MALDI-TOF-MS and MALDI-TOF/TOF-MS.

Spot	Accession number	Score	Peptide matched	Sequence coverage (%)	Mr/pI	Protein
3	gi|25989637	105	17/79	38	36232/7.00	lactate dehydrogenase [*E. acervulina*]
4	gi|145535758	72	26/93	22	155774/5.61	hypothetical protein GSPATT00019908001 *(P. t)*
6	gi|156097314	65	19/83	19	159966/6.14	6-phosphofructokinase, putative *(P. v)*
7	gi|118490906	154	12/37	50	27839/9.31	hypothetical protein (*E. tenella*)
8	gi|118490770	317	42/96	40	84827/8.27	hypothetical protein (*E. tenella*)
10	gi|124804156	64	16/86	19	84877/9.22	hypothetical protein PF11_0247 *(P .f)*
11	gi|50400241	230	32/100	69	48458/5.62	Enolase (*E. tenella*)
13	gi|25989639	136	22/100	54	35399/6.54	lactate dehydrogenase (*E. tenella*)
16	gi|118397285	79	21/96	27	126785/7.55	hypothetical protein TTHERM_00947400 *(T. t)*
20	gi|401829	427	46/76	56	70814/5.16	heat-shock protein *[E. acervulina]*
21	gi|145517049	71	16/91	35	46979/9.32	hypothetical protein GSPATT00012541001 *(P. t)*
22	gi|401829	86	19/98	27	70814/5.16	heat- shock protein *[E. acervulina]*
23	gi|401829	605	66/98	67	70814/5.16	heat-shock protein *[E. acervulina]*
24	gi|50400241	260	30/89	64	48458/5.62	Enolase (*E. tenella*)
26	gi|124054715	215	17/50	66	13343/5.92	actin depolymerizing factor (*E. tenella*)
28	gi|1037176	269	39/78	29	77516/5.22	immunoglobulin heavy chain binding protein (*E. tenella*)
31	gi|118379641	66	17/88	20	106631/6.01	hypothetical protein TTHERM_00348310 *(T. t)*
32	gi|118377447	74	16/92	22	87148/6.14	hypothetical protein TTHERM_01207560 *(T. t)*
33	gi|4033429	112	22/92	43	58238/5.95	Pyruvate kinase (PK) (*E. tenella*)
34	gi|145494414	69	10/96	42	22764/9.18	hypothetical protein GSPATT00006303001 *(P. t)*
35	gi|118359856	77	14/94	23	70549/8.46	polyadenylate-binding protein 2 *(T. t)*
36	gi|4033429	146	24/72	46	58238/5.95	Pyruvate kinase (PK) (*E. tenella*)
38	gi|145519139	77	25/93	18	192289/5.98	hypothetical protein GSPATT00013486001 *(P. t)*
39	gi|124805406	70	11/97	34	37233/9.54	mitochondrial phosphate carrier protein *(P. f)*
40	gi|145498869	76	15/96	38	47850/10.02	hypothetical protein GSPATT00036807001 *(P. t)*
41	gi|50400241	132	23/100	48	48458/5.62	Enolase (*E. tenella*)
43	gi|21542248	214	33/94	62	50377/4.78	Tubulin beta chain (Beta-tubulin) (*E. tenella*)
44	gi|156098346	67	10/52	14	123864/9.42	hypothetical protein PVX_091055 *(P. v)*
45	gi|50400241	204	26/63	59	48458/5.62	Enolase (*E. tenella*)
46	gi|50400241	197	26/67	59	48458/5.62	Enolase (*E. tenella*)

Note: P. t: Paramecium tetraurelia strain d4-2; T. t: Tetrahymena thermophila SB210; P. f: Plasmodium falciparum 3D7; P. v: Plasmodium vivax SaI-1.* common protein in two stages

Thirteen spots were identified as known proteins of *E. tenella*. A group of proteins shown as antigenic proteins on immunoblots was identified as enolase and pyruvate kinase (PK), which may be involved in glycolysis. Enolases were detected in Spots 11, 24, 41, 45 and 46. PKs with an MW of 58 kDa were also detected in Spots 33 and 36. Other proteins, such as lactate dehydrogenase (Spot 13), known to be involved in glycolysis, actin depolymerizing factor (Spot 26), immunoglobulin heavy chain binding protein (Spot 28), and tubulin beta chain (Spot 43) were detected as immunogenic proteins of *E. tenella*. In addition, two hypothetical proteins of *E. tenella* were identified on Spots 7 and 8 which showed immunogenic with chicken anti-*E. tenella* serum. However, their functions were unknown.

Seventeen spots were identified as homologous proteins to other protozoa. Proteins on Spots 4, 21, 34, 38 and 40 were similar to hypothetical proteins of *Paramecium tetraurelia* (*P. tetraurelia*) strain d4-2. Proteins on Spots 16, 31 and 32 were found to be homologous to hypothetical proteins of *Tetrahymena thermophila* (*T. thermophila*) SB210. The protein on Spot 35 was identified as similar to polyadenylate-binding protein 2 of *T. thermophila* SB210. The proteins on Spots 10 and 39 were identified as hypothetical protein and mitochondrial phosphate carrier protein of *Plasmodium falciparum* (*P. falciparum*, PfmpC) 3D7, respectively. A 6-phosphofructokinase and a hypothetical protein of *Plasmodium vivax* (*P. vivax*) SaI-1 were also identified for the proteins on Spots 6 and 44, respectively. The proteins on Spots 20, 22 and 23 matched the heat-shock protein (HSP70), and the protein on Spot 3 matched the lactate dehydrogenase of *Eimeria acervulina* (*E. acervulina*).

### Identification of proteins of unsporulated *E. tenella* using the *E. tenella* protein sequence database

The results obtained by searching against the *E. tenella* protein database using the ‘Mascot in-house’ search engine (version 2.1) indicated that the proteins on 12 spots were unknown proteins of *E. tenella*. After comparison of amino acid sequences with those in the NCBI databases, 9 spots were found to be significantly homologous to other apicomplexan parasites, except Spots 14, 19 and 30 (Table 2).

**Table 2:** Identification of unsporulated *Eimeria tenella* oocyst proteins by *E. tenella* protein database from *E. tenella* genome assembly using ‘Mascot in-house’ and then amino acid sequence BLAST against non-redundant sequence databases (NCBI).

Spot	Accession number	Score	Peptide matched	Sequence coverage (%)	Mr/pI	Related contig	*E. tenella* genome assembly	Contig BLAST against NCBI database
2	XXX12652.tmp3	91	14/64	43	36760/7.59	Contig12652	complement (60072..61332)	
	gb|AAK20420.1							glyceraldehyde-3-phosphate dehydrogenase (*T. g*)
	ref|XP-001348772.1							glyceraldehyde-3-phosphate dehydrogenase (*P. f 3D7*)
5	XXX2043.tmp6	200	25/78	42	32616/8.14	Contig2043	complement (13545..17007)	
	emb|CAH03718.2							putative secretory protein *(T. a)*
9	XXX2256.tmp1	90	14/69	27	44109/6.53	Contig2256	complement (2339..7012)	
	gb|EAN34194.1							pyruvate dehydrogenase E1 component beta subunit, mitochondrial, putative *(T. p)*
12	XXX4578.tmp1	265	37/87	52	77965/8.48	Contig4578	complement (414..7001)	
	gb|AAC47725.3							myosin-B *(T. g)*
	ref|XP-766628.1							myosin B *(T. p strain Muguga)*
14	XXX6171.tmp6	204	27/80	30	54407/4.97	Contig6171	complement (38747..40753)	––––––––––
15	XXX12652.tmp25	239	35/96	40	55757/6.46	Contig12652	complement (7093..9803)	
	gb|ABB00911.1							eukaryotic translation initiation factor 5 (*T. g*)
17	XXX2989.tmp10	84	16/96	38	59063/6.62	Contig2989	complement (41668..45596)	
	gb|EDO05336.1							fumarate hydratase, putative *(B. b)*
18	XXX607.tmp12	228	49/93	26	174714/8.27	Contig607	complement (49066..58084)	
	gb|EDL46529.1							SNF2 family N-terminal domain containing protein *(P. v)*
19	XXX1454.tmp24	71	10/86	60	33007/ 4.46	Contig1454	complement (127070..127972)	––––––––––-
27	XXX12511.tmp24	76	14/98	55	27814/6.31	Contig12511	complement (12140..13529)	
	gb|EAL35019.1							proteasome subunit (*C. h TU502)*
29	XXX3535.tmp1	327	39/65	44	93367/8.44	Contig3535	complement (3785..8777)	
	gb|AAC47724.1							myosin-A (*T. g*)
30	XXX6171.tmp6	204	27/80	30	54407/4.97	Contig6171	complement (38747..40753)	––––––––––-

Note: *T. g: Toxoplasma gondii; P. f 3D7: Plasmodium falciparum 3D7; T. a: Theileria annulata; T. p: Theileria parva; T. g: Toxoplasma gondii; T. p strain Muguga: Theileria parva strain Muguga; B. b: Babesia bovis; P. v: Plasmodium vivax; C. h TU502: Cryptosporidium hominis TU502.*

The amino acid sequence of Spot 2 shared similarity of 76% and 74% to the glyceraldehyde-3-phosphate dehydrogenase of *Toxoplasma gondii* (*T. gondii*) and *P. falciparum* 3D7, respectively. For Spots 5 and 9, the amino acid sequences showed similarities of 52% and 68% to the putative secretory protein and pyruvate dehydrogenase E1 component beta subunit of mitochondria from *Theileria annulata* (*T. annulata*) and *Theileria parva* (*T. parva*), respectively. Similarly, the amino acid sequence of Spot 12 shared similarity of 43% and 35% to myosin B from *T. parva* strain Muguga and *T. gondii*, respectively. The protein on Spot 15 showed high homology to eukaryotic translation initiation factor 5 (62% identity) of *T. gondii*. The protein on Spot 17 matched 65% identity to fumarate hydratase of *Babesia bovis* (*B. bovis*). The protein on Spot 18 was close to SNF family N-terminal domain containing protein (52% identity) of *P. vivax*.

**Figure 3:**
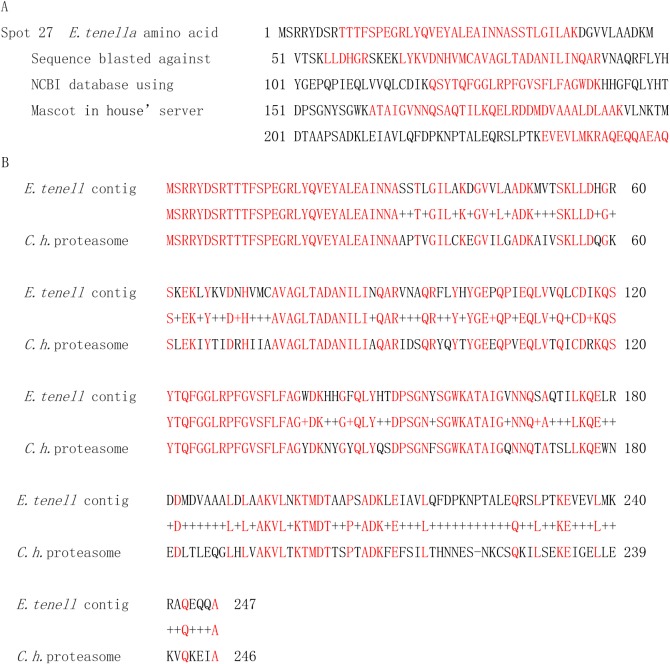
Spot 27's amino acid sequence homologous to *Cryptosporidium hominis* proteasome searched against the *E. tenella* genome using the ‘Mascot in-house’ server. A: *E. tenella* amino acid sequences obtained by MALDI-TOF-MS were searched using BLAST against the NCBI database. Deduced amino acid sequences on Spot 27 were aligned with the deduced sequence from the *E. tenella* genome Contig 12511: complements (12140…13529) are in bold red script. B: Alignment of the predicted amino acid sequence from Spot 27 (A) with the *C. hominis* proteasome subunit. The alignments of sequences are in bold red script.

The proteins on Spots 27 and 29 showed high homology to proteasome subunit (64% identity) and myosin A (59% identity) of *Cryptosporidium hominis* (*C. hominis*) TU502 (Fig. 3) and *T. gondii,* respectively. Spots 14, 19 and 30 were neither identified nor related to known parasite proteins. Detailed results are listed in Table 2.

## Discussion

At least seven *Eimeria* species parasitizing chickens have been reported [34]. Among them, *E. tenella* is the most virulent species in chickens [27]. *E. tenella* has a complicated life cycle with extracellular invasive stages that infect host cells in the ceca and intracellular multiplication stages [15]. After three asexual multiplication stages in the chicken cecum, the parasites undergo sexual differentiation to produce unsporulated oocysts and shed into feces of infected chickens [19].

To our knowledge, this study is the first report on unsporulated oocyst soluble proteins of *E. tenella* by an immunoproteomics method. In this study, the map showed the presence of many abundant spots between pI 3-10 in Fig. 1 and about 656 spots were separated on a 2D gel.

Database search results of the immunogenic proteins indicated that several different spots were matched to the same protein. This was probably attributed to the isoforms produced by post-translational proteolytic processing and modification, the cleavage of alkaline regions or phosphorylation of multiple residues. Similar results have been described in other reports [20,39].

Immunogenic proteins identified included: lactate dehydrogenase, enolase, HSP70, actin depolymerizing factor (ADF), immunoglobulin heavy chain binding protein (BiP), PK, tubulin beta chain (Beta-tubulin), hypothetical proteins and other homologous proteins from other Apicomplexa parasites or protozoa. Moreover, enolase, lactate dehydrogenase, and Beta-tubulin were detected in the other stages including sporozoite [11] and the second merozoite stage [24], and showed immunogenicity.

Enolase and PK were identified, which seems relevant to an adaptation of the metabolism in the anaerobic development of the intracellular stage as glycolytic enzymes [14,22,30]. De Venevelles et al. found the two enzymes as antigens in the sporozoite stage of *E. tenella* [11]. All these results would further support the theory that enolase may be an important immunogenic protein of *E. tenella*.

Beta-tubulin, as a subcellular structural protein, was also identified from unsporulated *E. tenella* oocysts. In other experiments, this protein was shown to induce immunity protective against natural infection [21].

The protein on Spot 29 was identified as homologous to myosin A of *T. gondii*. The head domain of myosin A of *T. gondii* demonstrated excellent conservation of the ATP and actin-binding domains, as well as other motifs consistently conserved among all myosins [3,5,18]. Myosins are actin-dependent molecular motors that play important roles in muscle contraction, cell motility and organelle transport [6]. ADF was identified as a specific immunogenic protein as Spot 26 in the unsporulated oocyst stage. ADF plays an important role in remodeling the actin cytoskeleton and contributes greatly to the invasion of host cells by the apicomplexan parasite [25,40].

Several spots (20, 22 and 23) were identified as heat-shock proteins by their homologies with the HSP70 of *E. acervulina*. Parasite invasion often induced an increase in the synthesis of parasite HSP70, either in response to an increase in the host's temperature or due to stress from the invasive process [26,28].

The proteins on Spot 28 matched the BiP of *E. tenella* in unsporulated oocysts. As a protein in the endoplasmic reticulum (ER), BiP plays a role in the assembly and the folding of proteins [12,17]. Two hypothetical proteins of *E. tenella* (Spots 7 and 8) were found to be highly immunogenic, but we were not able to find their function in the databases of NCBI and UniProt [23].

The protein on Spot 39 of unsporulated oocysts matched PfmpC. The PfmpC as a mitochondrial carrier protein belonged to one of nine members of the mitochondrial carrier family present in *P. falciparum*, including an ATP/ADP exchanger and a di/tri-carboxylate exchanger, probably involved in transport of TCA cycle intermediates across the mitochondrial membrane [4,16].

The protein on Spot 2 was found to be highly similar to glyceraldehyde-3-phosphate dehydrogenase of *T. gondii* (76% identity) and *P. falciparum* (71% identity). It is a tetrameric NAD-binding enzyme involved in glycolysis and glyconeogenesis [13]. Similarly, the Spot 5 protein was highly homologous to putative secretory protein of *T. annulata* (52% identity). The Spot 15 protein was similar to eukaryotic translation initiation factor 5 (62% identity) of *T. gondii*, but function was still unclear. The Spot 17 protein was similar to fumarate hydratase (65% identity) of *B. bovis*. The fumarate hydratase (fumarase) is a component of the citric acid cycle, and also engages in the reductive pathway from oxaloacetate to succinate during anaerobic growth [6].

A proteasome subunit of *C. hominis* TU502 showed high similarity to the Spot 27 protein. The high homology (64% identity) indicated the close relation between the two species. Proteasome, as a complex of proteins, could exert the function of the central enzyme of nonlysomal protein degradation in both the cytosol and nucleus [41].

Comparing the substances with those identified in Liu et al. (2009) [24], lactate dehydrogenase, enolase and heat-shock proteins were found in both studies. Some surface secretory proteins, such as surface antigen and microneme proteins, and regulatory proteins, such as 14-3-3 protein, were recognized by the sera isolated from chickens infected experimentally with *E. tenella* in the second-generation merozoite stage of *E. tenella*. In addition, some glycolytic enzymes, such as 6-phosphofructokinase and PK, and microfilamentous proteins, such as actin depolymerizing factor, were identified in the unsporulated oocyst stage of *E. tenella*.

In conclusion, this is the first report on the immunoproteomic profiling of unsporulated *E. tenella* oocysts. Forty-six spots were identified as immunogenic proteins. These new findings enrich knowledge of the immunogenic proteins of the unsporulated oocyst stages of *E. tenella*. The newly identified antigens are of great interest in terms of understanding pathogen-host interactions and in the search for novel vaccine targets. Further investigations into the characterization and functions of the immunogenic proteins would be beneficial to clarify the mechanisms of immune responses, immune evasion, immunopathology, and in the development of vaccines against *Eimeria*.
